# Visualizing traumatic brain injuries

**DOI:** 10.7554/eLife.65676

**Published:** 2021-02-02

**Authors:** Marc Ekker

**Affiliations:** Department of Biology, University of OttawaOttawaCanada

**Keywords:** traumatic brain injury, tauopathy, post-traumatic seizures, epilepsy, in vivo models, neurotrauma, Zebrafish

## Abstract

Zebrafish larvae models can be used to study the link between seizures and the neurodegeneration that follows brain trauma.

**Related research article** Alyenbaawi H, Kanyo R, Locskai LF, Kamali-Jamil R, DuVal MG, Bai Q, Wille H, Burton AE, Allison WT. 2021. Seizures are a druggable mechanistic link between TBI and subsequent tauopathy. *eLife* **10**:e58744. doi: 10.7554/eLife.58744

Traumatic brain injuries are a leading cause of death and disability in younger people, as well as an important risk factor for neurodegenerative diseases and dementia in older adults. They can be caused by direct physical insults, whiplash or shockwaves such as those produced by explosions (Cruz-Haces et al., 2017). Yet traumatic events can also have effects beyond the immediate death and damage to neurons. In particular, they can disrupt a protein known as Tau, which normally maintains the stability of many neuronal cells. When this happens, an abnormal, hyperphosphorylated version of Tau accumulates in cells and spreads throughout the central nervous system by turning healthy Tau proteins into the harmful variant (Johnson et al., 2013; Ojo et al., 2016; Zanier et al., 2018). This accumulation is the hallmark of illnesses known as tauopathies, which include Alzheimer’s disease and a progressive brain condition found in athletes who experience regular head blows.

Scientists need accessible animal models in which they can easily observe and manipulate the proliferation of abnormal Tau proteins after a brain trauma. Rat and mouse models exist, but they are expensive and not well suited to visualizing what is happening inside the brain. Now, in eLife, Ted Allison (University of Alberta) and colleagues – including Hadeel Alyenbaawi (Alberta and Majmaah University) as first author and other researchers in Alberta and Pittsburgh – report new zebrafish larvae models for both tauopathies and traumatic brain injuries (Alyenbaawi et al., 2021).

The first model is formed of transgenic, ‘Tau-GFP reporter’ zebrafish in which the spread of the abnormal protein can be directly observed. To achieve this result, Alyenbaawi et al. genetically manipulated the animals so that their neurons would carry a reporter version of Tau that is fused with a green fluorescent protein (or GFP). As the larvae are transparent, their nervous system and the fluorescent Tau are easily visible. The fish were then injected with abnormal mice Tau proteins, causing the reporter Tau to aggregate into mobile ‘puncta’ – small dots which are a hallmark of tauopathies. More puncta were observed when extracts from brains with Tau-linked conditions were injected into the larvae, rather than the normal proteins.

Alyenbaawi et al. also devised a simple and inexpensive zebrafish model for traumatic brain injury. They put the larvae inside a closed syringe, and dropped a weight onto the plunger, creating a shockwave to mimic blast injuries in humans. Three days in a row of this regimen creates conditions reminiscent of those faced in repetitive sports injury. In the Tau-GFP reporter larvae, the shockwave treatment led to fluorescent puncta in the brain and spinal cord, consistent with traumatic brain injuries leading to Tau pathologies (Figure 1). Similarly, past reports have shown that healthy mice developed a Tau-linked condition when they received brain extracts from conspecifics that experienced traumatic brain injuries (Zanier et al., 2018).

**Figure 1.**
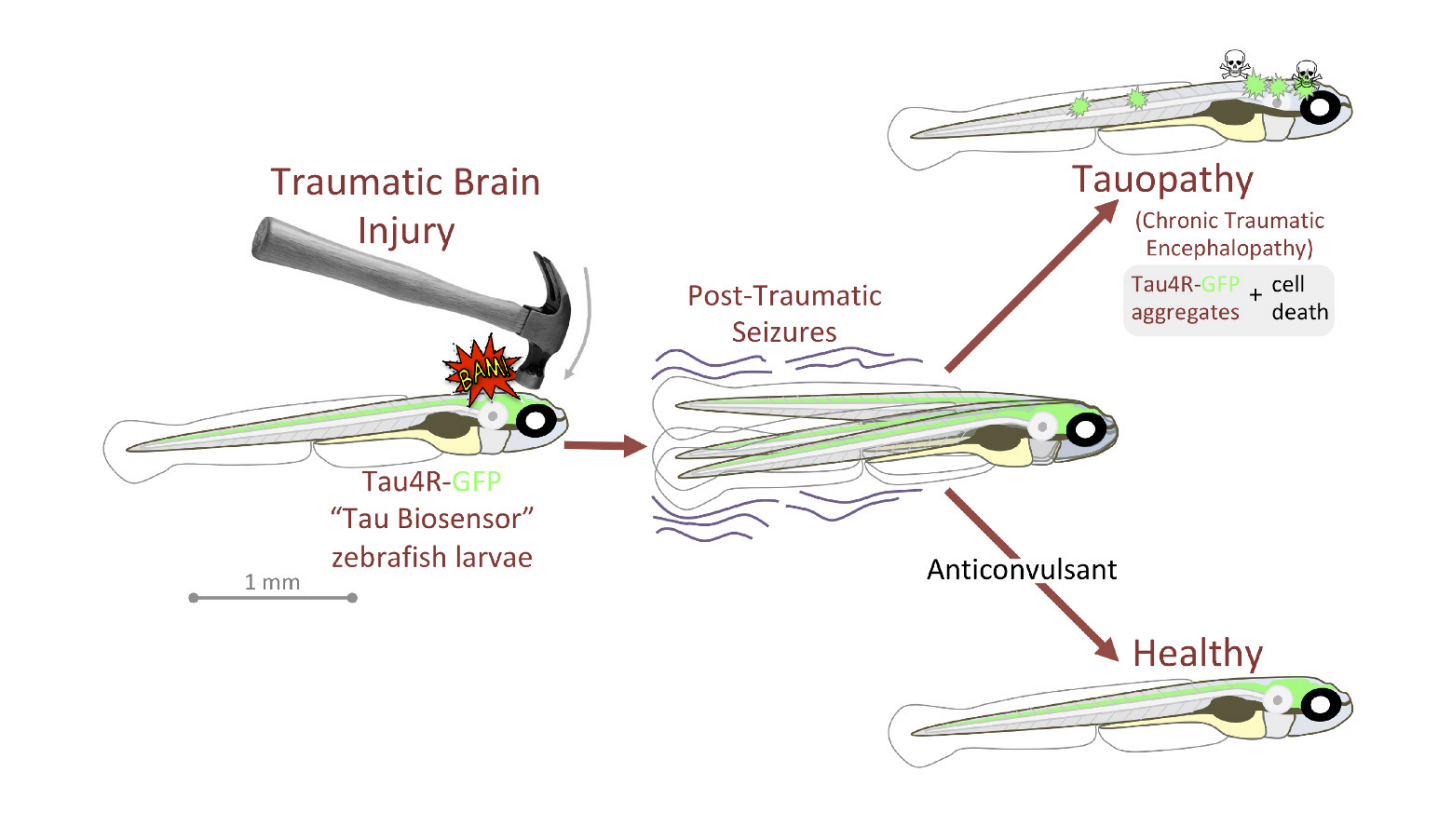
Traumatic brain injury results in seizures and Tau-linked conditions in zebrafish larvae. Zebrafish larvae with neurons that carry Tau proteins fused with a fluorescent reporter (Tau4R-GFP) are subjected to a brain injury (left). Many then experience seizures, and without treatment they develop a Tau-linked condition in which the proteins aggregate and the neurons die (top right). Larvae that receive anticonvulsants are protected to a certain extent against seizures and the Tau-linked illness (bottom right).

Image credit: Figure based on figure 7 of Alyenbaawi et al., 2021 (CCBY 4.0).

In humans, epileptic seizures appear in over half of traumatic brain injury victims, and especially in those who have received a blast injury; these episodes may initiate or exacerbate the progression of Tau-linked conditions. In zebrafish, the traumatic brain injury larvae also developed seizure-like behaviors, with the intensity of the seizures being positively correlated to the spread of abnormal Tau. Drugs that promoted or stopped seizures respectively increased or decreased the extent of the Tau-linked condition, suggesting that anticonvulsants could help to manage brain traumas in the clinic (Figure 1).

Alyenbaawi et al. carefully identified the limitations of their models, observing for instance that the Tau-GFP reporter could spread in larvae even when the animals did not receive anomalous Tau proteins. This may result from relatively high levels of the Tau reporter in the transgenic animals, outlining the importance of controlling the expression levels of the transgene.

Apart for a recent model which used ultrasound, very few methods have been available so far to simulate traumatic brain injury in zebrafish (Cho et al., 2020). This was particularly the case for larvae, despite these young animals having a more easily observable central nervous system, a high throughput, and an ethical advantage compared to adults. The models developed by Alyenbaawi and colleagues thus constitute a welcome addition to understand the mechanisms associated with traumatic brain injury.
